# α-Mangostin Disrupts the Development of *Streptococcus mutans* Biofilms and Facilitates Its Mechanical Removal

**DOI:** 10.1371/journal.pone.0111312

**Published:** 2014-10-28

**Authors:** Phuong Thi Mai Nguyen, Megan L. Falsetta, Geelsu Hwang, Mireya Gonzalez-Begne, Hyun Koo

**Affiliations:** 1 Institute of Biotechnology, Vietnam Academy of Science and Technology, Hanoi, Vietnam; 2 Center for Oral Biology, University of Rochester Medical Center, Rochester, New York, United States of America; 3 Biofilm Research Labs, Levy Center for Oral Health, Department of Orthodontics, School of Dental Medicine, University of Pennsylvania, Philadelphia, Pennsylvania, United States of America; University of Oklahoma Health Sciences Center, United States of America

## Abstract

α-Mangostin (αMG) has been reported to be an effective antimicrobial agent against planktonic cells of *Streptococcus mutans*, a biofilm-forming and acid-producing cariogenic organism. However, its anti-biofilm activity remains to be determined. We examined whether αMG, a xanthone purified from *Garcinia mangostana* L grown in Vietnam, disrupts the development, acidogenicity, and/or the mechanical stability of *S. mutans* biofilms. Treatment regimens simulating those experienced clinically (twice-daily, 60 s exposure each) were used to assess the bioactivity of αMG using a saliva-coated hydroxyapatite (sHA) biofilm model. Topical applications of early-formed biofilms with αMG (150 µM) effectively reduced further biomass accumulation and disrupted the 3D architecture of *S. mutans* biofilms. Biofilms treated with αMG had lower amounts of extracellular insoluble and intracellular iodophilic polysaccharides (30–45%) than those treated with vehicle control (*P*<0.05), while the number of viable bacterial counts was unaffected. Furthermore, αMG treatments significantly compromised the mechanical stability of the biofilm, facilitating its removal from the sHA surface when subjected to a constant shear stress of 0.809 N/m^2^ (>3-fold biofilm detachment from sHA vs. vehicle-treated biofilms; *P*<0.05). Moreover, acid production by *S. mutans* biofilms was disrupted following αMG treatments (vs. vehicle-control, *P*<0.05). The activity of enzymes associated with glucan synthesis, acid production, and acid tolerance (glucosyltransferases B and C, phosphotransferase-PTS system, and F_1_F_0_-ATPase) were significantly inhibited by αMG. The expression of *manL*, encoding a key component of the mannose PTS, and *gtfB* were slightly repressed by αMG treatment (*P*<0.05), while the expression of *atpD* (encoding F-ATPase) and *gtfC* genes was unaffected. Hence, this study reveals that brief exposures to αMG can disrupt the development and structural integrity of *S. mutans* biofilms, at least in part via inhibition of key enzymatic systems associated with exopolysaccharide synthesis and acidogenicity. αMG could be an effective anti-virulence additive for the control and/or removal of cariogenic biofilms.

## Introduction

Many infectious diseases in human are caused by virulent biofilms, including oral diseases [1]. Among them, dental caries continues to be one of the most ubiquitous and costly biofilm-dependent diseases throughout the world [2], [3]. For organisms associated with caries development, the production of an extracellular polysaccharide (EPS)-rich biofilm matrix, acidification of the milieu, and the maintenance of acidic pH microenvironment in close proximity to the tooth enamel are major controlling virulence factors linked with the pathogenesis of the disease. Current therapeutic approaches to control pathogenic oral biofilms fall short; the search for new/improved agents may lead to more efficacious anti-caries therapies [4]–[6]. Natural products are currently regarded as potentially promising sources for new bioactive agents that may function to suppress these key virulence attributes that are associated with the establishment and maintenance of cariogenic biofilms [5].

The assembly of cariogenic biofilms results from complex interactions that occur between specific oral bacteria, the products they produce, host saliva and dietary carbohydrates, all of which occurs on pellicle-coated tooth surfaces [7], [8]. *Streptococcus mutans* has been recognized as one of the key etiologic agents associated with the initiation of dental caries, although additional organisms may contribute to its pathogenesis [9]. Sucrose is considered the primary catalyst for caries development, as it serves as a substrate for the production of both EPS and acids. *S. mutans* can effectively form cariogenic biofilms when sucrose is available, because this bacterium rapidly synthesizes EPS (from sucrose) through the activity of exoenzymes (e.g. glucosyltransferases; Gtfs) [8]. At the same time, *S. mutans* produces acid and is highly aciduric, allowing it to tolerate and continue to produce acids in low pH microenvironments, while readily adapting to acidic and other environmental stresses [10]–[14].

EPS synthesis via *S. mutans-*derived Gtfs is critical for cariogenic biofilm formation, since the glucans produced by the secreted exoenzymes (present in the pellicle-coated tooth and on bacterial surfaces) promote local bacterial accumulation, while embedding bacteria in a diffusion-limiting matrix. These processes create highly cohesive and adhesive biofilms that are firmly attached to surfaces and are difficult to remove [15]–[18]. At the same time, the EPS-rich matrix shelters resident organisms from antimicrobial and other inimical influences [18]–[20]. In parallel, sugars (in addition to sucrose) are fermented by *S. mutans* and other acidogenic bacteria ensnared within the biofilm matrix, creating acidic microenvironments across the three-dimensional (3D) architecture and at the surface of attachment [18], [21], [22]. Acidification of the milieu favors growth of aciduric organisms, further enhancing EPS production and ensuring biofilm accrual and localized acid-dissolution of the enamel in areas where biofilm is present and pH is low [18], [23]. Therefore, using bioactive agents that target EPS-mediated biofilm assembly and acidogenicity could disrupt the pathogenesis of dental caries in a highly effective and precise manner.

Plants are valuable sources of new bioactive compounds to combat dental caries, because they produce a wide variety of secondary metabolites, many of which have been found to have biological properties against oral pathogens *in vitro* (as reviewed in Jeon et al. [5]). *Garcinia mangostana* L. (Guttiferae) is a widely cultivated fruit tree in Southeast Asian nations, including Thailand, Sri Lanka, The Philippines, and Vietnam [24]. The pericarp of *G. mangostana* has been used in traditional medicine to treat a variety of infections. Experimental studies have demonstrated that xanthone derivatives are the major bioactive substances, exhibiting antioxidant, antitumor, anti-inflammatory, and antimicrobial activities [24]–[26].

Our previous work showed that αMG exhibits antimicrobial activity against planktonic *S. mutans* cells via multiple actions, particularly reducing acid production by disrupting the membrane of this organism [27]. However, the question as to whether this agent is capable of compromising the ability of *S. mutans* to develop biofilms using a clinically relevant treatment regiment (brief topical exposures) remains to be elucidated. Therefore, the aim of the present study was to investigate the potential effectiveness of topical applications of αMG and its biological actions against *S. mutans* biofilm formation on saliva-coated apatitic surfaces.

## Materials and Methods

### Extraction and isolation of α-mangostin

**Figure 1:**
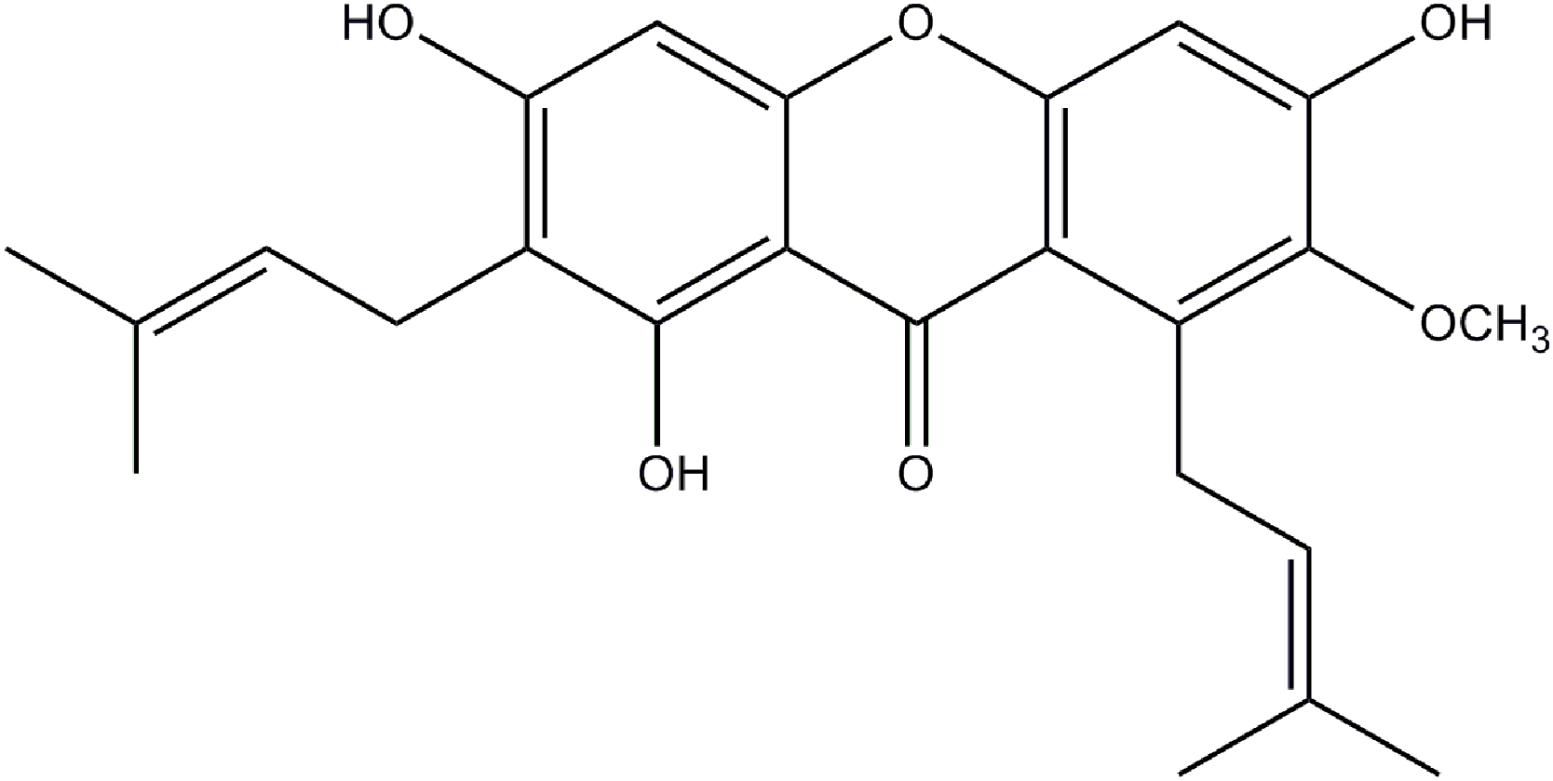
Chemical structure for αMG. Molecular formula: C_24_H_26_O_6_. Molecular weight: 410.466.

*Garcinia mangostana L* is a fruit plant widely available in the south of Vietnam. The dried powder of samples of *Garcinia mangostana* peels collected from Binhduong province (south of Vietnam) was used in this study. No specific permission for collection of *G. mangostana* is required for this location because it is not an endangered or protected species. Ethanolic extracts of *G. mangostana* were prepared for the initial step of αMG isolation. The dried powder of *G. mangostana* peels collected from the South of Vietnam were extracted with ethanol at room temperature, followed by an evaporation of solvent to give a dark brown gummy residue. This residue was taken up in water followed by extraction with *n*-hexane to produce the most bioactive fractions. The *n*-hexane fraction was then evaporated and dried under reduced pressure. Further separation was performed using silica gel column chromatography (Merck Kieselgel 60, 70–230 mesh) by eluting with *n*-hexane – ethyl acetate – methanol (6∶3∶0.1, by volume) and 10 mL volumes of eluant were collected in test tubes. The aliquots of each fraction were subjected to thin-layer chromatography (60 F254, 1 mm plate, Merck) in a solvent system containing toluene – ethyl acetate – acetone – formic acid (5∶3∶1∶1, by volume). Partially purified αMG was recovered from the active fractions and then further separated by silica gel column chromatography (Merck Kieselgel 60, 70–230 mesh) and eluting with *n*-hexane – chloroform – ethyl acetate – methanol (4∶1∶0.5∶0.3, by volume), yielding a single compound, αMG, as yellow crystals. The purity of αMG was examined by high-pressure liquid chromatography connected with mass spectrometry (LCMSD- Trap-SL Mass spectra, Agilent 1100, Palo Alto, California). The chemical structure (Fig. 1) of αMG was determined using nuclear magnetic resonance (Bruker Avance 500 spectrometer, Germany).

The compound at concentration of 100, 150 and 200 µM was dissolved in 25% ethanol, which was also used as a vehicle control; treatments with 25% ethanol did not affect the viability of cells of *S. mutans* in a biofilm when compared to untreated controls. The pH of the treatment solution was maintained at 5.8±0.2, based on the observation that αMG activity is best at acidic pH [27].

### Preparation and treatment of the biofilm

**Figure 2:**
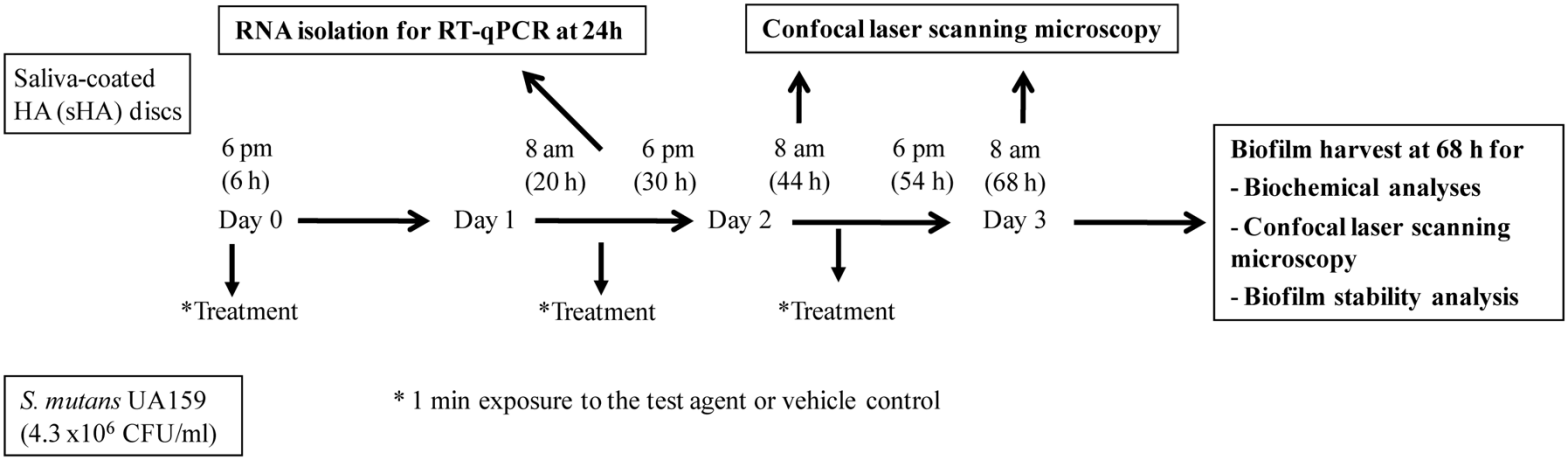
The experimental design for the treatment and analysis of biofilms of *S. mutans.* The clinical conditions of typical exposure of exogenously introduced therapeutic agents in the mouth were simulated by applying the test agent twice daily for brief exposures (60 s) at early/initial formation of the biofilm (6 h). Subsequently, the biofilms were treated twice at 8 am (20 h-old) and 6 pm (30 h-old) with two more additional treatments the following day (8 am; 44 h-old and 6 pm; 54 h-old).

*S. mutans* UA159 (ATCC 700610), a proven virulent-cariogenic strain selected for genomic sequencing, was used in this study. Biofilms of *S. mutans* were formed on saliva coated hydroxyapatite (sHA) surfaces (12.7 mm in diameter, 1 mm in thickness, Clarkson Chromatography Products Inc., South Williamsport, PA), as previously described [28]. The biofilms were grown in ultra-filtered (10 kDa MW cut-off membrane; Prep/Scale, Millipore, MA) buffered tryptone-yeast extract broth (UFTYE; 2.5% tryptone and 1.5% yeast extract with the addition of 4.35 g/L of potassium phosphate and 1 g/L of MgSO_4_·7H_2_O, pH 7.0) with 1% sucrose at 37°C and 5% CO_2_. Briefly, *S. mutans* cells in exponential growth phase were inoculated into UFTYE and applied to wells containing sHA discs placed vertically in a custom-made holder. Biofilms were allowed to form on sHA discs and were treated for the first time with the test agents or vehicle control after 6 h of development. Subsequently, the biofilms were treated at 8 am (20 h-old) and 6 pm (30 h-old), with two more additional treatments the following day (8 am; 44 h-old and 6 pm; 54 h-old). The biofilms were exposed to the treatments for 60 s, dip-washed in sterile saline solution (0.89% w/v NaCl) to remove excess agents, and then transferred to fresh culture medium [29], [30]. The biofilm was analyzed after 44 h and 68 h using confocal microscopy to examine the effects on the overall 3D architecture after receiving the initial topical treatments (Figure 2). At 68 h, the biofilms were removed, homogenized and subjected to biochemical analysis as detailed previously [28]. Briefly, biomass was assessed with an aliquot of the homogenized suspension centrifuged at 10,000 *g* for 10 min at 4°C, and the cell pellet was washed twice with water, then dried in the dry oven at 105°C for 24 h and weighed [28]. The water soluble and insoluble exopolysaccharides (EPS), and intracellular iodophilic polysaccharides (IPS) were extracted and quantified via colorimetric assays [28]. The total number of viable cells in each of the biofilms was determined by counting colony forming units (CFU), while total protein was quantified via ninhydrin assays as descrbed in Koo et al. [28]. Furthermore, the pH of the culture media of treated and untreated biofilms was monitored every 2 hours with an Orion pH electrode attached to an Orion 290 A+ pH meter (Thermo Fisher Scientific).

### Confocal microscopy of biofilms

The overall effect of topical applications of αMG on the 3D architecture and the spatial distribution of EPS and bacterial biomass within intact biofilms was assessed using confocal fluorescence imaging [18]. Briefly, 2.5 µM Alexa Fluor 647-labeled dextran conjugate (10,000 MW; absorbance/fluorescence emission maxima 647/668 nm; Molecular Probes Inc., Eugene, OR) was added to the culture medium during the formation and development of *S. mutans* biofilms. The fluorescently-labeled dextran serves as a primer for Gtf-mediated glucan synthesis and can be simultaneously incorporated during EPS matrix synthesis over the course of biofilm development, but does not stain the bacterial cells at the concentrations used in the study. The bacterial cells in the biofilms were labeled with 2.5 µM SYTO 9 green-fluorescent nucleic acid stain (480/500 nm; Molecular Probes Inc., Eugene, OR) using standard procedures [18]. Laser scanning confocal fluorescence imaging of the biofilms was performed using an Olympus FV 1000 two-photon laser scanning microscope (Olympus, Tokyo, Japan) equipped with a 10 X (0.45 numerical aperture) water immersion objective lens. Each biofilm was scanned at 5 randomly selected positions on the microscope stage and the confocal image series were generated by optical sectioning at each of these positions. Three independent experiments were conducted. The step size of z-series scanning was 2 µm. The confocal images were analyzed using software for simultaneous visualization of EPS and bacterial cells within intact biofilms [18], [31], [32]. Amira 5.4.1 software (Visage Imaging, San Diego, CA) was used to create 3D renderings of each structural component (EPS and bacteria) to examine the architecture of the biofilm.

### Determination of mechanical stability of biofilms

The mechanical stability of the biofilms treated with or without αMG was compared using a custom built device (detailed information is in Figure S1). Biofilms were exposed to constant shear stress of 0.809 N/m^2^ for 10 min, which is capable of removing *S. mutans* biofilm from sHA surface; such shear stress was determined as a threshold for >50% removal of untreated *S. mutans* biofilms from saliva-coated HA surfaces using our model. Shear stress at the biofilm surface was produced by shear flow generated via rotating paddle, and estimated based on Reynolds number of the flow (turbulent flow) and the surface friction using Blasius formula (Supplemental information). The amount of biofilm dry-weight (biomass) before and after application of shear stress for each condition (vehicle- and αMG-treated) was determined. Then, the percentage of biofilm that remained on sHA disc surface was calculated. All experiments were performed in quadruplicates in three distinct experiments.

### Gtf Docking Analyses

In the present study, different bioinformatics tools and databases were used. The crystal structure of glucosyltransferases C (GtfC) from the dental caries pathogen *Streptococcus mutans* is available in the Protein Data Bank (PDB) and was used as a receptor for docking of the αMG compound (ligand) using HEX software. Since the crystal structure of GtfB is not yet available, Phyre server [33] was used to predict ligand sites. HEX has been reported as an interactive molecular graphic program. It calculates protein-ligand docking, assuming that the ligand is rigid and then superimposes pairs of molecules using only their 3D shapes [34], [35]. In addition, it uses Spherical Polar Fourier (SPF) correlations, increasing the speed of the calculations, and it also has integrated graphics software to view the final result [35]–[38]. PDB was used to download the crystal structure of glucansucrase from the dental caries pathogen *Streptococcus mutans* (http://www.rcsb.org/pdb/home/home.do). PubChem Compound was used for retrieving the 3D-structure of α-mangostin (http://www.ncbi.nlm.nih.gov/pccompound). MarvinSketch software was utilized for obtaining the α-mangostin structure in a PDB format (http://www.chemaxon.com/products/marvin/marvinsketch/), and the HexServer (HEX 6.9 software) was accessed for calculating and displaying protein-ligand docking (http://hexserver.loria.fr/). The parameters used for docking included: Correlation type (Shape only), FFT mode (3D fast life), Grid dimension (0.6), Receptor range (180), Ligand range (180), Twist range (360), and Distance range (40) were used.

### Determination of Gtf activity

GtfB and GtfC were obtained from recombinant strains carrying the appropriate genes as detailed elsewhere [34]. Strain *S. milleri* KSB8 harboring the *gtf*B gene transformed from *S. mutans* GS-5 and *S. mutans* WHB 410 construct expressing *gtf*C gene only were used. The GtfB and GtfC enzymes (E.C. 2.4.1.5) were prepared from culture supernatants and purified to near homogeneity by hydroxyapatite column chromatography. The purified Gtfs (1–1.5 U) were mixed with the test compound and incubated with a [U-^14^C-glucose]-sucrose substrate (0.2 µCi/ml; 200.0 mmol of sucrose per liter, 40 µmol of dextran 9000 per liter, and 0.02% sodium azide in adsorption buffer consisting of 50 mM KCl, 1.0 mM KPO_4_, 1.0 mM CaCl_2_, and 0.1 mM MgCl_2_, pH 6.5) to a final concentration of 100 mmol of sucrose per liter (200 µl final volume) at 37°C with rocking for 4 h. For the vehicle-control, the same reaction was carried out with 25% ethanol (v/v) replacing the test agent solutions. Glucosyltransferase activity was measured by incorporation of [U-^14^C-glucose] from labeled sucrose into glucans [34]. The radiolabelled glucans were quantified by scintillation counting.

### F-ATPase and phosphotransferase system (PTS) assays

F-ATPase and PTS activity of treated biofilm cells were determined as described by Belli and Marquis [39] and Phan et al. [40]. Biofilms were homogenized and centrifuged at 4°C, and then biofilm pellets from each sample were resuspended in 2.5 ml of 75 mM Tris-HCl buffer (pH 7.0) with 10 mM MgSO_4_. Toluene (250 ul) was added to each biofilm cell suspension prior to vigorous vortex mixing and incubation for 5 min at 37°C. Each suspension was then subjected to two cycles of freezing in a dry ice-ethanol bath and thawing at 37°C. Permeabilized biofilm cells were harvested by centrifugation. They were then resuspended in 1.0 ml of 75 mM Tris-HCl buffer (pH 7.0) with 10 mM MgSO_4_. The suspension was quickly frozen in a dry ice-ethanol bath and stored at −70°C for F-ATPase and PTS assays. F-ATPase activity was determined as described by Belli and Marquis [39]. The F-ATPase reaction is initiated by the addition of 30 µl of 0.5 M ATP (pH 6.0). Samples of 50 µl were removed and assayed for inorganic phosphate liberated from cleavage of ATP with reagents from American Monitor Co. (Indianapolis, IN) [39]. Phosphotransferase system (PTS) activity was assessed in terms of pyruvate production from phosphoenolpyruvate in response to glucose addition. Pyruvate was assayed by use of lactic dehydrogenase and measurements of the change in absorbance of 340 nm light associated with oxidation of NADH [39].

### Reverse transcription quantitative PCR (RT-qPCR)

RT-qPCR was performed to evaluate the expression of the *gtfB, gtfC, atpD*, and *manL* genes. Biofilms were treated as described in the Figure 2. RNA was extracted and purified using standard protocols optimized for biofilms [41]. The RNA integrity numbers (RIN) of purified samples used for RT-qPCR were determined by microcapillary electrophoresis on an Agilent 2100 Bioanalyzer (Agilent Technologies, Santa Clara, CA). Purified RNA samples (RIN≥9) were stored in RNase-free water at −80°C. cDNAs were synthesized from 1 µg of purified RNA using a BioRad iScript cDNA synthesis kit (Bio-Rad Laboratories, Inc., Hercules, CA). RNA samples without reverse transcriptase were included as a negative control. The resulting cDNAs and negative controls were amplified by a MyiQ qPCR detection system with iQ SYBR Green supermix (Bio-Rad Laboratories, Inc., CA, USA) and specific primers. When Taqman probes were available, cDNAs and controls were amplified using a Bio-Rad CFX96 system (Bio-Rad Laboratories). The 16S rRNA primers/TaqMan probes were run separately, and primers/TaqMan probes for other specific targets were combined and used in a multiplex setting. For reactions with only one TaqMan probe (used for target 16S rRNA), the iQ Supermix (BioRad) was used. For multiplex reactions (*gtfB*, *gtfC*) and (*atpD*, *manL*) the iQ Multiplex Powermix (BioRad) were employed. Standard curves were used to determine the relative number of cDNA molecules, which were normalized to the relative number of 16S rRNA cDNA in each sample, as described previously [42]. 16S rRNA served as a reference gene [43]. These values were used to determine the fold-change between each treated sample and the vehicle control. The MIQE guidelines [44] were followed for quality control of the data generated and for data analysis. The gene expression profile was determined 4 h after the topical treatment at 20 h (Figure 2), to evaluate the impact of αMG on *S. mutans* within the accumulated biofilms post-treatment. This time point represents the most active period of the biofilm development using our model, and was selected based on our biochemical data and previous studies on the dynamics of the *S. mutans* transcriptome during biofilm formation on sHA and in response to topically applied agents [43], [45].

### Statistical analyses

Data are presented as the mean ± one standard deviation (SD). Pair-wise comparisons were made between test and control using Student’s t-test. Statistical analysis was performed using JMP (version 3.1; SAS Institute, Cary, NC). The level of significance was set at 5%.

## Results and Discussion

### αMG disrupts the accumulation and acidogenicity of *S. mutans* biofilms

In our experiment, *S. mutans* biofilms were initially treated with α-mangostin (αMG) at concentrations of 100, 150, and 200 µM (Table S1) based on bioactivity against planktonic *S. mutans* cells [27] and solubility in the vehicle system. We selected a concentration of 150 µM αMG, because it was as effective as 200 µM in reducing the overall biofilm development and acid production.

The data in Table 1 indicate that treatments with 150 µM αMG significantly reduced the accumulation of *S. mutans* biofilms on saliva-coated apatitic surfaces, which resulted in less biomass (dry-weight) and less total protein compared to the vehicle control (*P*<0.05). The viability of the biofilms was not significantly impacted by the treatments. Nevertheless, short-term topical applications (one-minute exposure, twice daily) significantly reduced the amount of polysaccharides in the biofilms (Table 1). The amount of insoluble exopolysaccharides (EPS) was drastically reduced, while the soluble EPS content was unaffected by αMG treatments. The data suggest that GtfB and GtfC, which are largely responsible for the synthesis of insoluble glucans in the biofilm matrix [8], could be targeted by αMG; while possibly having limited effects on the activity of GtfD (involved for soluble glucan synthesis). Interestingly, the amount of intracellular iodophilic polysaccharides (IPS), a glycogen-like storage polymer [46], was significantly disrupted by treatments with the agent.

**Table 1:** *Streptococcus mutans* UA159 biofilm composition after treatments with 150 µM αMG.

Biofilm composition	Vehicle	150 µM αMG
**Dry weight** (mg/biofilm)	4.73±0.41	3.00±0.45*
**Protein** (mg/biofilm)	2.85±0.38	1.67±0.19*
**Soluble EPS** (µg/biofilm)	326.6±37.2	229.7±91.0
**Insoluble EPS** (µg/biofilm)	1112.1±151.7	356.9±49.0*
**IPS** (µg/biofilm)	188.3±17.8	79.9±22.6*
**CFU/biofilm**	2.77E+08±5.98E+07	2.25E+08±5.50E+07

Data are expressed as the mean ± one standard deviation. For each parameter, values marked with an asterisk are significantly different from that for the vehicle control (n = 8; *P*<0.05, pair-wise comparison using Student’s t test).

Altogether, the biochemical changes inflicted by αMG may affect the matrix assembly and 3D biofilm architecture, which could disrupt the mechanical stability and adhesive strength of the treated biofilms.

### αMG compromises the 3D architecture and mechanical stability of *S. mutans* biofilms

**Figure 3:**
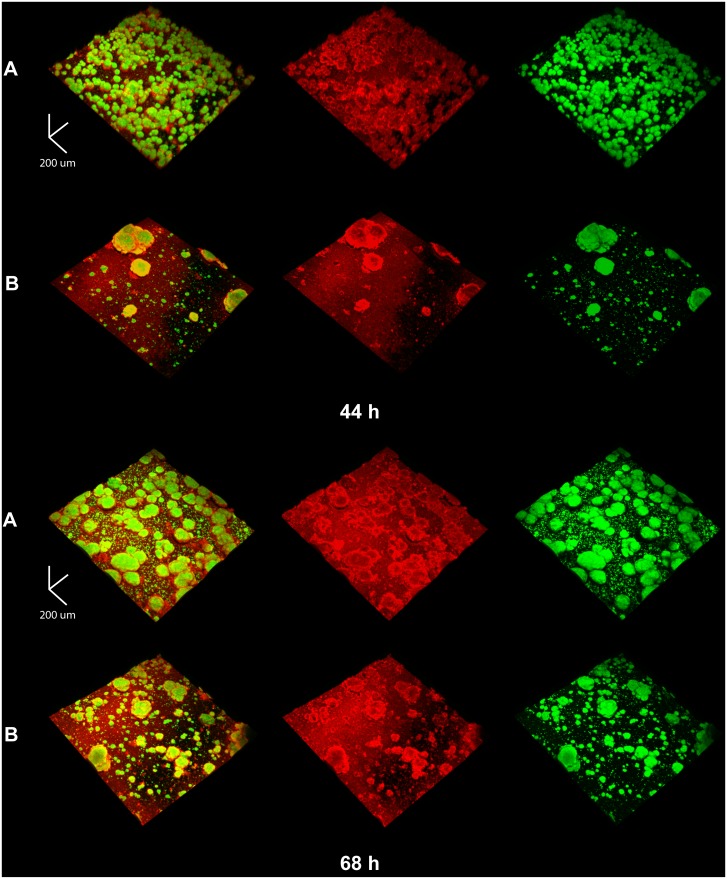
Representative 3D rendered images of 44 h and 68 h-old *S. mutans* biofilms following topical treatments. Biofilms were treated with the vehicle control in panel A and with 150 µM αMG in panel B. The EPS channel is in red, while bacterial cells are in green. Scale bars = 100 µm. Biofilms were formed on hydroxyapatite discs (sHA) in the presence of 1% (wt/vol) sucrose, and treated with test agents twice daily.

Confocal images revealed a marked impairment in the development of an insoluble EPS-matrix (in red), as well as the defective formation of bacterial clusters or microcolonies (in green) following αMG treatment, particularly at 44 h (Figure 3). The few microcolonies detected in the αMG-treated biofilms at 44 h visually appear to be larger than those treated with vehicle-control, suggesting that microcolony development was not completely inhibited. Nevertheless, the defective biofilm assembly resulted in an altered 3D architecture (at 68 h) characterized by sparsely distributed microcolonies (with many areas on the sHA surface that were devoid of such structures), as well as a less developed EPS matrix, compared to vehicle-treated biofilms. These findings agree well with our biochemical data showing a significant reduction in the insoluble EPS content.

These structural changes may affect the stability of the biofilms treated with αMG and facilitate mechanical clearance of biofilms. The mechanical stability of biofilms appears to be dependent on the exopolysaccharide content, as EPS binds the cells together while strengthening their cohesiveness [15], [47]–[50]. Furthermore, glucans enhance *S. mutans* adhesive strength, while the development of multi-microcolony aggregates via EPS-cell adhesions provides structural integrity to *S. mutans* biofilms [16], [18]. Thus, we hypothesized that the disruptive effects of αMG could facilitate biofilm removal and/or detachment. We investigated the impact of αMG on mechanical stability of *S. mutans* biofilms using a custom-built shear-inducing device (Figure S1).

**Figure 4:**
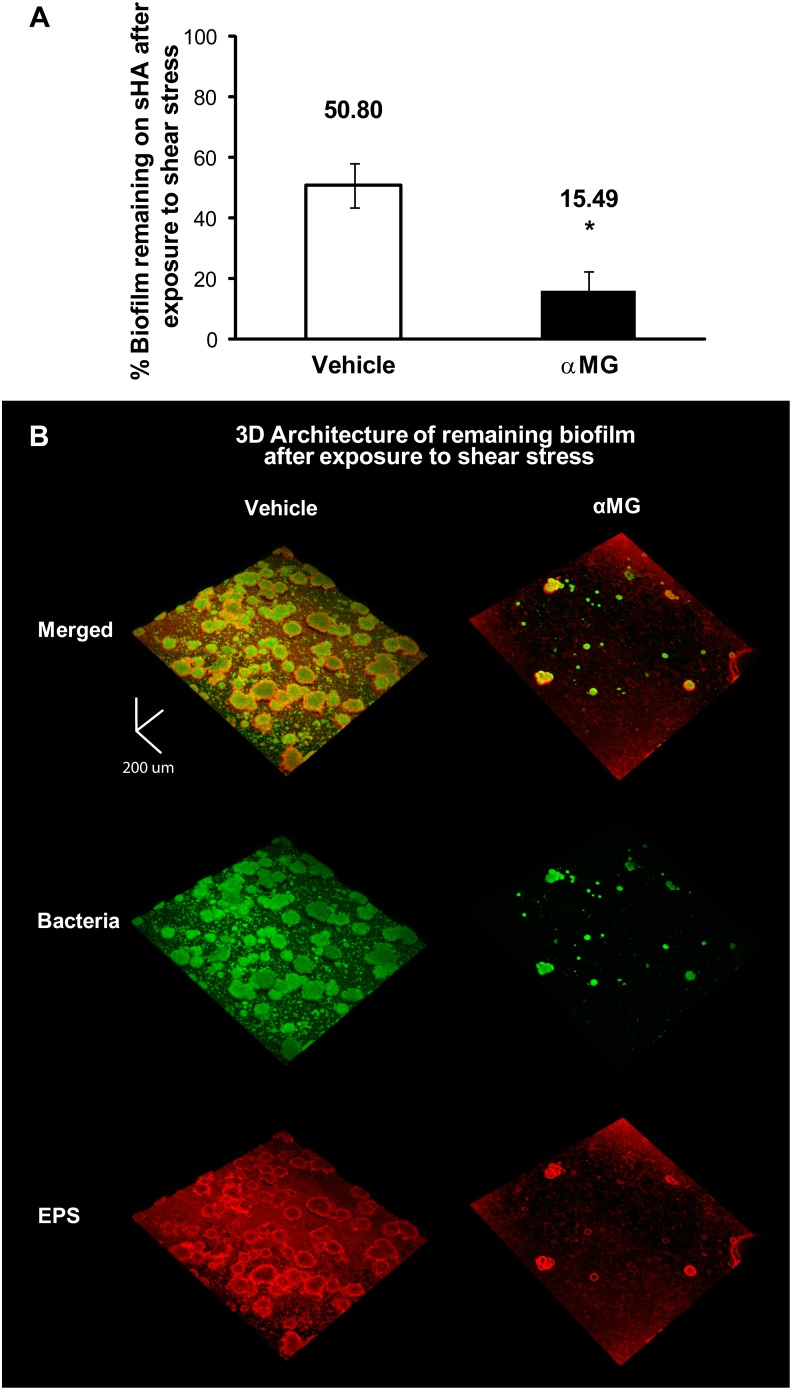
Biofilm mechanical stability following topical treatments. Panel A depicts the percentage of biomass of vehicle- or αMG-treated biofilms that remained on sHA after exposure to shear stress. The amount of biofilm dry-weight (biomass) before and after application of shear stress for each condition (vehicle- and αMG-treated) was determined, and the percentage of biofilm that remained on sHA disc surface was calculated. Data are expressed as the mean ± one standard deviation. Values are significantly different from that for the vehicle control (n = 12; *P*<0.05, pair-wise comparison using Student’s t test). Panel B shows representative 3D rendered images of treated-biofilms of *S. mutans* after shearing. The EPS channel is in red, and the bacterial cells are in green. Scale bars = 100 µm.

The ability of treated-biofilms to withstand mechanical removal under shear stress was determined by measuring the amount of biofilm biomass (dry-weight) that remained on the sHA after shearing (Figure 4A). We observed that αMG-treated biofilms were more effectively removed from the sHA surface (84.51% removal) than those treated with vehicle-control (49.2%; *P*<0.05) when subjected to shear stress, indicating that the mechanical stability of the biofilms was compromised by αMG. Indeed, confocal images of αMG-treated biofilms show that most of the bacterial biomass and EPS was removed, while vehicle-treated biofilms show numerous EPS-enmeshed bacterial microcolonies still attached on the sHA surface (Figure 4B). Clearly, the data demonstrate that alterations in the EPS-matrix and microcolony assembly resulted in significantly less adherent biofilms, which facilitated their mechanical clearance from the sHA surface when exposed to shear force. By reducing the production of insoluble EPS, αMG treatments could affect optimal microcolony formation and surface anchoring as well as the cell-matrix cross-linking forces and the overall viscoelasticity, which have been shown to be critical for weakening the biofilm structure [18], [49], [51]. Further studies shall elucidate how αMG affects the adhesion forces and rheological properties of the biofilms locally.

### αMG inhibits GtfB and GtfC activity

Previous studies have shown that extracellular glucans produced by GtfB and GtfC enzymes play vital, yet distinct roles in the formation of cariogenic biofilms and are essential in the pathogenesis of dental caries (as reviewed in Bowen and Koo [8]). The glucans synthesized by GtfC assemble the initial EPS layers on the sHA surface, which provide enhanced binding sites for *S. mutans* colonization and accumulation [52], [53]. Conversely, the highly insoluble and structurally rigid glucans formed by GtfB embed the cells, contributing to the scaffolding of the 3D EPS-rich matrix [18]. The accumulation of Gtf-derived EPS and bacteria cells mediates the construction of EPS-enmeshed microcolonies that are firmly anchored to the apatitic surface [16]–[18], [54]. Here, we examined whether αMG is capable of inhibiting the activity of purified GtfB and GtfC enzymes, which could explain the defective assembly and attachment of the treated biofilms observed in this study. Since there is no previous data on Gtf inhibition by αMG, we initially examined the likelihood of the agent to bind Gtfs using *in silico* docking studies.

**Figure 5:**
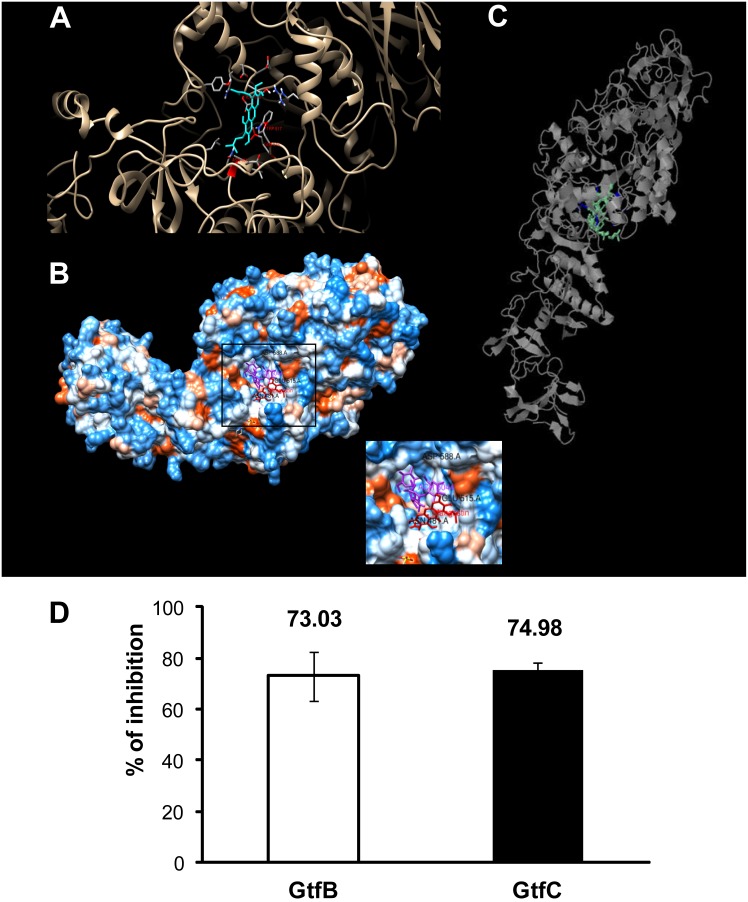
Snapshot of glucosyltransferase interaction with αMG compound and influence of 150 µM αMG on the activities of GtfB and GtfC. Panel A depicts the ribbon model of glucosyltransferase C (brown) docking α-mangostin (blue) using HEX-docking software Amino acids, such as Trp 517, Glu 515, Asp 588 and Asn 481 are interacting in the glucosyl binding site. Panel B depicts the surface model of glucosyltransferase C docking α-mangostin (red) and acarbose (purple) using HEX-docking software. Panel C depicts the glucosyltransferase B 3D ligand-binding site predicted model using Phyre Server. Panel D depicts Gtf activity of *S. mutans* cells when treated with αMG. The percentage of inhibition was calculated setting the vehicle control to 100% Gtf activity. Data are expressed as the mean ± one standard deviation. Values are significantly different from that for the vehicle control (n = 12; *P*<0.05, pair-wise comparison using Student’s t test).

Docking studies support the prediction of conformation and binding affinity for selected molecules against a given target protein [55]. Therefore, docking of αMG on Gtf was carried out to explore if/how this compound might interact with the enzymes. In our study, when the GtfC enzyme was docked with αMG, the energy value obtained by HEX software was −511.36 Kcal/mol, indicating a stable and strong binding between the two molecules [55]. The best docked structure, visualized by UCSF Chimera molecular modeling system version 1.8 (http://www.cgl.ucsf.edu/chimera/download.html), showed the interaction of four amino acids (Trp 517, Glu 515, Asp 588 and Asn 481) (Figure 5A and 5B). A previous report by Ito et al. [56] indicated that binding to Glu 515 compromised the acid/base catalyst function, while interaction with Trp 517 blocked the acceptor glycosyl moiety. These observations can explain the inhibitory properties shown by acarbose when bound to Gtf-SI [56]. As displayed in figure 5B, αMG and acarbose interact with Trp 517, which provides the main frame for the glycosyl acceptor binding site. Since the crystal structure of GtfB is not yet available, Phyre server [33] was used to predict ligand sites. The obtained results highlighted the presence of hydrophobic amino acids Leu 356, Gln 35, Ala 409, Lys 408, Asn 410; Asp 878, Ser 880; Ser 884, Leu 882, Tyr 936, Phe 881; Asn 1026 and some other amino acids with electrically charged amino acids like Asp 838I (Figure 5C).

All amino acids mentioned above are found in catalytic or the glucan binding regions of both GtfB and GtfC [57]–[60], suggesting that the function of these enzymes could be affected by αMG. Indeed, the enzymatic activity of purified GtfB and GtfC was impacted by αMG as shown in Figure 5D. The test agent was highly effective in reducing glucan synthesis by both enzymes, displaying more than 70% inhibition (vs. vehicle control) at 150 µM, which agrees well with the in silico analysis, as well as the biochemical (reduction of insoluble EPS content) and confocal imaging (defective assembly of EPS-matrix and impaired microcolony formation) data of the αMG-treated biofilms.

### αMG affects acidogenicity of *S. mutans* biofilms, and disrupts F-ATPase and PTS activities

**Figure 6:**
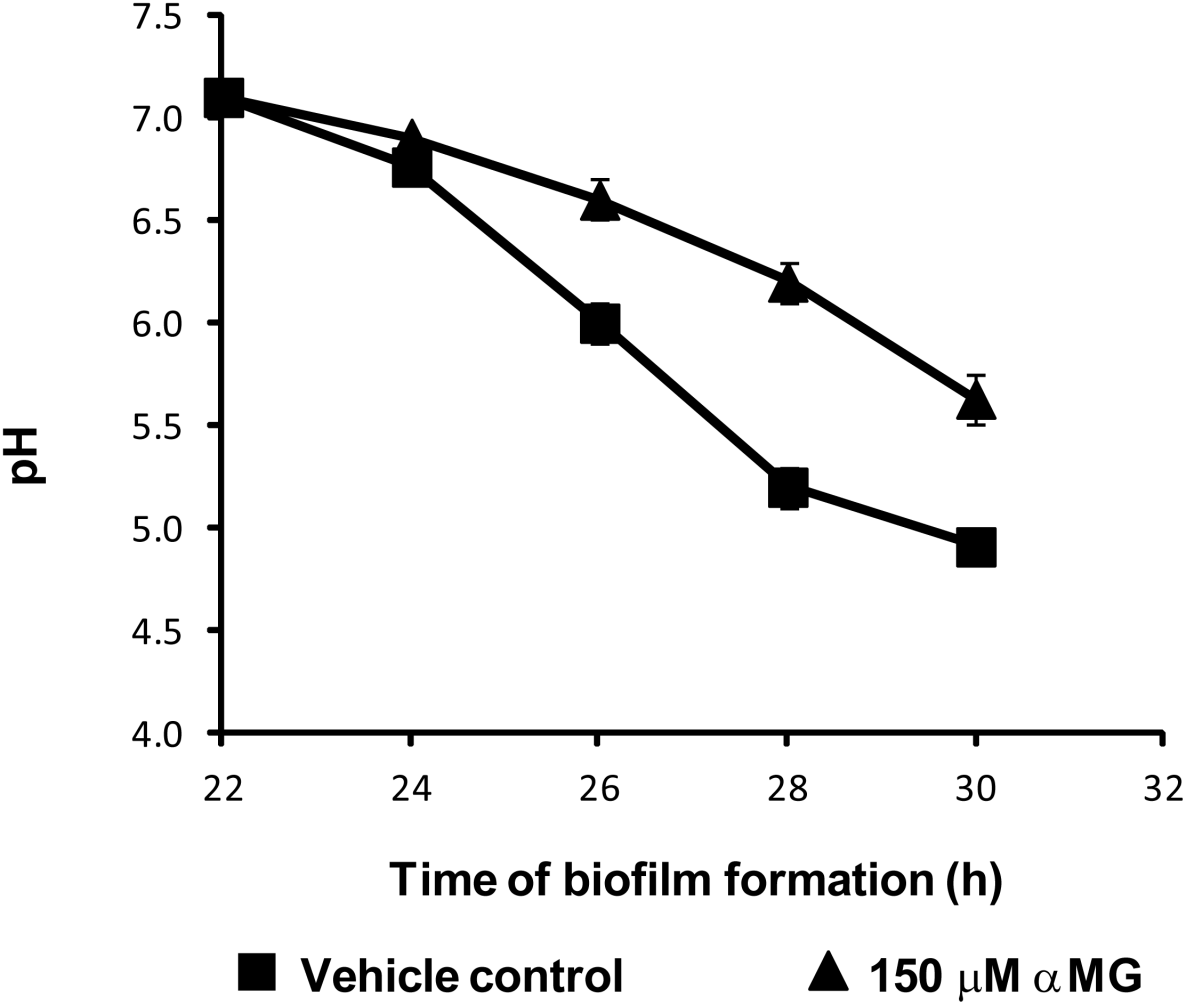
Effects of αMG on acid production by *S. mutans* UA159. Vehicle is represented by (▪), while 150 µM αMG is represented by (▴). Data are expressed as the mean ± one standard deviation for experiments run in triplicates in at least three separate experiments.

The biofilm EPS-matrix and microcolonies provide *S. mutans* with niches, where it survives and carries out glycolysis, even at low pH values, resulting in demineralization of the adjacent dental enamel [8], [39]. In addition to the deleterious effects on biomass accumulation and structural organization, αMG also affected *S. mutans* biofilm acidogenicity following topical applications of the agent (Figure 6). αMG reduced both the acid production and the acid tolerance of *S. mutans* biofilm cells as indicated in the pH-drop profile (Figure 6). The test agent sensitized the biofilm cells to acidification to the point that the final pH value was significantly higher (∼1 unit) than those treated with vehicle-control (P<0.05), suggesting that there may be disturbances in the activity of the proton-translocating membrane F-ATPase [27], [39].

**Figure 7:**
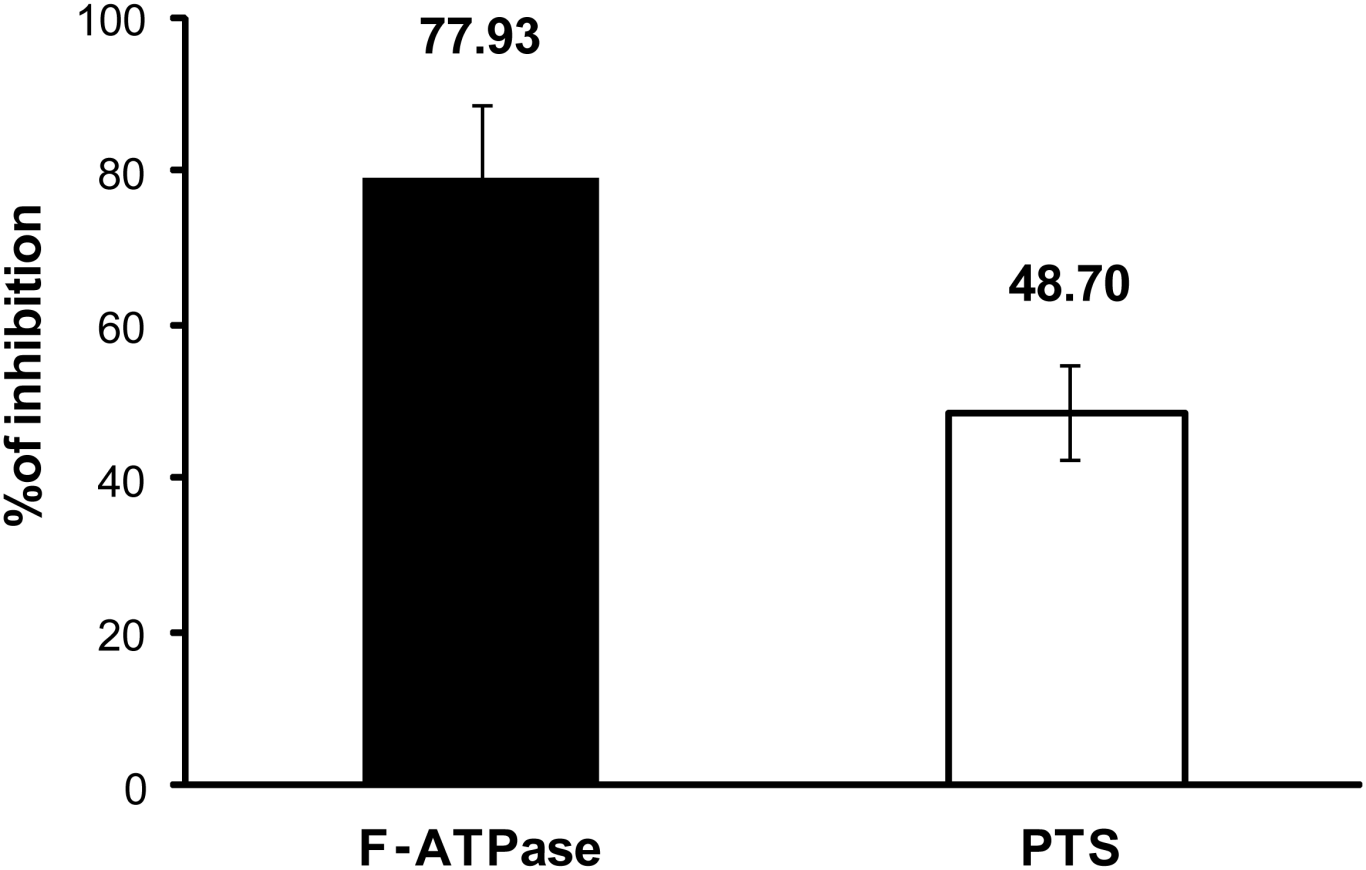
Effects of αMG on ATPase and PTS activities of *S. mutans* UA159. The percentage of inhibition was calculated setting the vehicle control to 100% enzymatic activity. Data are expressed as the mean ± one standard deviation. Values are significantly different from that for the vehicle control (n = 9; *P*<0.05, pair-wise comparison using Student’s t test).

A previous study has shown that αMG was particularly effective in inhibiting the activity of F(H^+^)-ATPase and PTS [27], which are critical for acid production and acid-tolerance and to ensure the optimum function of glycolysis by *S. mutans* within biofilms [61]. However, the assays were conducted with *S. mutans* grown in the planktonic phase. In this study, we examined the F-ATPase and PTS activity of biofilm cells following the treatment with αMG. The membrane-bound F-ATPase (H^+^-translocating ATPase) is considered the primary determinant for acid tolerance [61]. During glycolysis, protons are pumped out of the cell by F-ATPase to help maintain ΔpH across the cell membrane, preventing acidification of the cytoplasm, which would typically inhibit intracellular enzymes [39]. Furthermore, under certain conditions, it also generates ATP for *S. mutans* growth and persistence [62]. The data in Figure 7 show that the F-ATPase activity was strongly inhibited by αMG with nearly 80% inhibition following topical treatments.

Conversely, sugar uptake by oral streptococci occurs primarily by means of the PTS system [63]. In this system, phosphoenolpyruvate (PEP), provided by glycolysis, is cleaved by Enzyme I and the phosphate group is transferred to a general phosphocarrier protein, HPr, which in turn acts as a phosphate donor to membrane-bound Enzyme II [63]. Thus, the system catalyzes the transfer of phosphate to an incoming sugar and translocation of it across the cell membrane to yield a sugar phosphate in the cytoplasm, at which point sugar is metabolized via glycolytic pathways to produce organic acids. As shown in Figure 7, the PTS activity of biofilms treated with αMG was also significantly inhibited (∼50% inhibition vs. vehicle-treated biofilms, P<0.05). Although the exact nature of αMG inhibition of the F-ATPase and PTS system found in this study remains to be determined using purified enzymes, our data suggest that αMG can affect *S. mutans* biofilms acidogenicity by disrupting the activity of these critical membrane-associated enzymes (albeit at concentrations of 3–5 times higher than those found against planktonic cells [27].

The inhibitory effects of αMG on F-ATPase and PTS could have additional impact on biofilm composition and virulence. Cytoplasmic acidification and reduction of sugar transport not only disrupts glycolytic acid production, but also the formation and accumulation of intracellular iodophilic polysaccharides (IPS) [46], which could explain at least in part the marked reduction of IPS in the treated biofilms (Table 1). The role of IPS in *S. mutans* virulence and dental caries in general has been clearly documented [64]–[66]. IPS provides *S. mutans* with an endogenous source of carbohydrates that can be metabolized when exogenous fermentable substrates have been depleted within the oral cavity [67]. As a result, IPS can help to promote the formation of dental caries by prolonging the exposure of tooth surfaces to organic acids and a concomitant lower fasting pH in the matrix of the plaque [65]. Thus, the inhibition of IPS accumulation by αMG could also contribute with the overall disruptive effects of the agent on *S. mutans* biofilms acidogenicity.

### αMG has limited effects on *gtfBC, atpD* and *manL* gene expression by *S. mutans* biofilms

**Figure 8:**
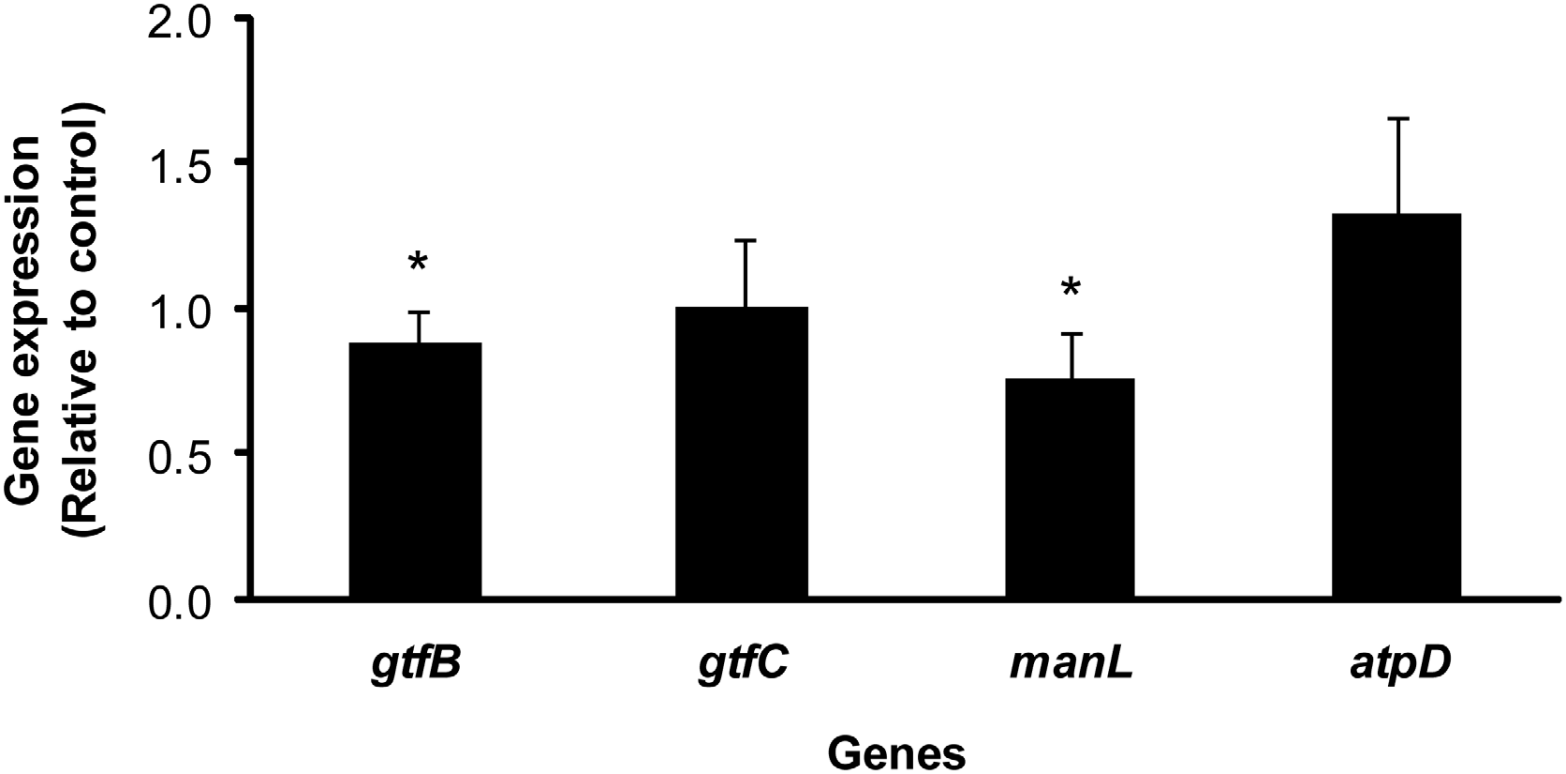
The expression of *S. mutans* genes *gtfB*, *gtfC*, *manL,* and *atpD* in biofilms. Fold changes ± one standard deviation. Values marked with asterisks are significantly different from that for the vehicle control (n = 8; *P*<0.05, pair-wise comparison using Student’s t test).

Treatment of biofilms with α-mangostin could inhibit insoluble EPS synthesis and glycolytic pH drop in either of the following two ways: i) reducing enzymatic function and/or ii) affecting transcription of the genes encoding these enzymes to reduce the amount of enzyme produced. Therefore, we profiled the transcription of *gtfB, gtfC*, *atpD* (encoding F-ATPase), and *manL* (encoding a key component of the mannose PTS). The expression profiles of these genes are shown in Figure 8. Overall, RT-qPCR analysis showed only a slight repression of *gtfB* and *manL* after treatment with αMG **(**P<0.05), while no significant effects were observed on *gtfC* and *atpD* expression, suggesting that the reduction in EPS biomass in treated biofilms may be largely due to the impact on enzymatic function (Figure 5 and 7).

Upon biofilm establishment, the resident microorganisms, encased in an EPS-rich matrix, are difficult to remove or treat, while a highly acidogenic and aciduric biofilm environment is created [20]. In this paper, we reported that topical application of α-mangostin (αMG) can disrupt some of the major virulence properties of *S. mutans* within biofilms, impairing further biofilm accumulation and acidogenicity, while facilitating mechanical clearance. Although previous studies have shown the biological actions of αMG against planktonic cells of *S. mutans* and other organisms, this is the first study demonstrating the antibiofilm effects of this promising phytochemical agent. Analysis of our data shows that αMG could affect biofilm development by *S. mutans* through at least three distinctive and yet interconnected ways: 1) disruption of insoluble EPS-matrix assembly at least in part by inhibiting GtfB and GtfC enzymatic activities, 2) compromising the mechanical stability, which may be linked to defective EPS production and impaired microcolony formation (thereby facilitating biofilm detachment from sHA surface), and 3) reducing acidogenicity by affecting IPS accumulation and the activities of the F-ATPase and PTS system. The results from this study indicate that GtfB and GtfC, as well as the F-ATPase and PTS enzymatic systems, are therapeutic targets of αMG.

In conclusion, our study demonstrated that the phytochemical αMG may represent a potentially useful anti-virulence additive for the control and/or removal of cariogenic biofilms. Having shown here that αMG exhibits significant bioactivity against *S. mutans* biofilms, further understanding of the molecular mechanisms of action of this agent as well as its effects on mixed-species cariogenic biofilm models are certainly warranted. Furthermore, cytotoxicity studies revealed that αMG is non-toxic and is generally regarded as safe [68]–[70]. Clearly, the efficacy of our treatment needs to be evaluated *in vivo* using a rodent model of dental caries.

## Supporting Information

Figure S1 **Biofilm mechanical strength testing device.** This supplementary material shows the design of the custom-built device to evaluate biofilm mechanical strength, and the principles of shear stress calculation.
(DOCX)

Table S1 **Effects of α-mangostin on biofilm accumulation by** ***S. mutans*** **UA159.**
(DOCX)
