# Supplementary material for: α-Mangostin Disrupts the Development of Streptococcus mutans Biofilms and Facilitates Its Mechanical Removal
Source: PLoS One. 2014 Oct 28;9(10):e111312. doi: 10.1371/journal.pone.0111312 (PMC4211880; doi:10.1371/journal.pone.0111312)
Supplement: Figure S1 — Biofilm mechanical strength testing device. This supplementary material shows the design of the custom-built device to evaluate biofilm mechanical strength, and the principles of shear stress calculation. (DOCX) [file pone.0111312.s001.docx]

**Supplemental Info: Shear-inducing device**

**Design of shear-induced biofilm mechanical strength tester** **(s-BMST)**

A shear-induced biofilm mechanical strength tester (s-BMST) was designed and machined to generate specific range of shear stress in a local machine shop at the University of Rochester. The schematic diagram of the s-BMST is presented in Fig. S1. Briefly, eight rods inserted from the aluminum lid contain each disc holder made of Delrin® acetal homopolymer resin (DuPont, DE). The shaft with paddle (50.8 mm × 80.5 mm) at the bottom is assembled with the lid. Each disc holder holds two 12.7 mm discs, and the discs are faced toward the paddle at a distance of 4.8 mm. The lid with disc holders and paddle is placed in a glass vessel. Shear stress is generated and adjusted by the paddle rotated by an overhead stirrer (Model BDC1850, Caframo, ON, Canada). The eight rods are fixed by a bottom plate made of Delrin® acetal homopolymer resin to minimize a wobble during shearing.


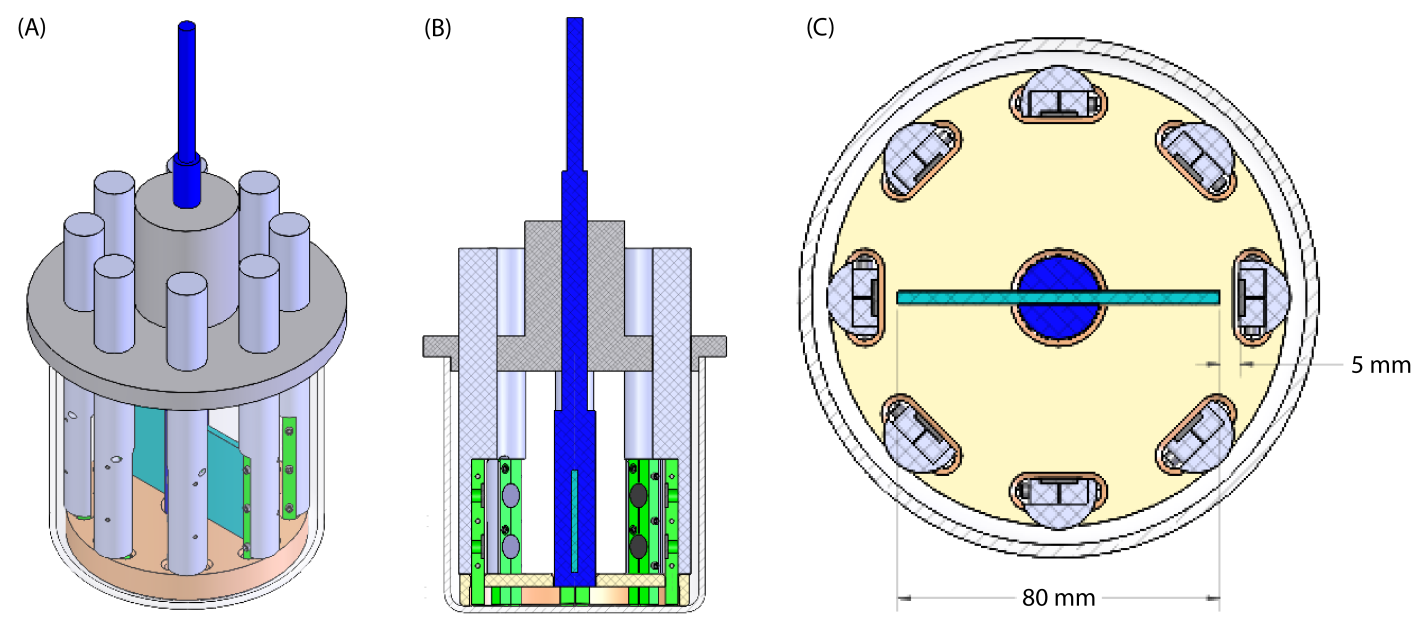


Fig. S1. Schematic diagram of shear-induced biofilm mechanical strength tester (s-BMST). (A) Bird view, (B) front view, and (C) floor view. Shear stress is generated by the rotation of the paddle, and adjusted by the RPM (revolutions per minutes).

**Estimation of shear stress at the disc surface**

The shear stress at the disc surface was estimated by modeling the s-BMST as two concentric cylinders, and assuming that the bulk fluid in the s-BMST has the properties of water at 20 °C. An inner cylinder of radius *R_I_* (40 mm) rotates at a constant speed *Ω* in an imaginary outer cylinder of radius *R_O_* (45 mm) (Fig. S1). In this study, the wall fluid shear stress at the disc surface was determined to be a turbulent flow by calculating Reynolds number (*Re*) as follows (Characklis and Marshall, 1990):

$Re= \frac{Ω\alpha R_{O}^{2}\rho}{\mu}$ (1)

where Ω is the rotational speed of inner paddle, α is the ratio inner to outer cylinder radius, ρ and µ are the density and viscosity of water at 20 °C, respectively.

Then, the surface friction (*f*) was calculated using the Blasius formula as follows (Darby, 2001):

$f=\frac{0.0791}{{Re}^{0.25}}$ (2)

Finally, shear stress at disc surface was estimated as follows (McCabe and Smith, 1976):

$\tau=\frac{f\rho v^{2}}{2}$ (3)

where $v^{2}=Ω^{2}R_{I}R_{O}$.

**References**

Characklis WG and Marshall KC. 1990. Biofilm process. In: Characklis WG and Marshall KC, editors. New York, NY: Wiley. p. 288

Darby R. 2001. Chemical engineering fluid mechanics. Second edition, revised and expanded ed. New York: Marcel Dekker, Inc.

McCabe WL, Smith JC. 1976. Unit operations of chemical engineering. New York: McGraw-Hill.
