# Supplementary material for: α-Mangostin Disrupts the Development of Streptococcus mutans Biofilms and Facilitates Its Mechanical Removal
Source: PLoS One. 2014 Oct 28;9(10):e111312. doi: 10.1371/journal.pone.0111312 (PMC4211880; doi:10.1371/journal.pone.0111312)
Supplement: Table S1 — Effects of α-mangostin on biofilm accumulation by S. mutans UA159. (DOCX) [file pone.0111312.s002.docx]

**Table S1. Effects of** αMG **on biofilm accumulation by *S. mutans* UA159**

| **Biofilm data** | **Vehicle** | **100 μM αMG** | **150 μM αMG** | **200 μM αMG** |
| --- | --- | --- | --- | --- |
| **Dry weight** (mg/biofilm) | 5.35 ± 0.34 | 4.35 ± 0.81 | 3.91 ± 0.20 | 3.77 ± 0.61 |
| **Protein** (mg/biofilm) | 3.23 ± 0.32 | 2.29 ± 0.42 | 2.17 ± 0.10 | 2.28 ± 0.51 |
| **Soluble EPS** (µg/biofilm) | 455.42 ± 37.92 | 362.33 ± 49.00 | 348.36 ± 21.69 | 353.50 ± 40.92 |
| **Insoluble EPS** (µg/biofilm) | 1893.03 ± 183.74 | 1352.36 ± 238.80 | 1103.70 ± 38.29 | 1112.63 ± 205.46 |
| **IPS**  (µg/biofilm) | 211.96 ± 11.15 | 142.49 ± 25.36 | 116.68 ± 4.83 | 132.39 ± 24.49 |
| **Final pH after**  **30 hour** | 4.78 | 5.20 | 5.49 | 5.65 |

Biofilms at initial formation (6 h) were treated with 150 μM α−mangostin (αMG) twice daily for brief exposures (60 s). 68 h-old biofilms were harvested for biochemical analysis. Data are expressed as mean ± one standard deviation. For each parameter (except pH), the values from 150 and 200 µM αMG are significantly different from that for the vehicle control (n = 4; *P* < 0.05, ANOVA, comparison for all pairs using Tukey test), while the differences between the values from 150 and 200 µM αMG are not statistically significant (*P* > 0.05).
